# Catabolism of *Dictyophora indusiata* Polysaccharide and Its Impacts on Gut Microbial Composition during In Vitro Digestion and Microbial Fermentation

**DOI:** 10.3390/foods12091909

**Published:** 2023-05-06

**Authors:** Yun-Xuan Zhao, Ling Huang, Ding-Tao Wu, Jie Li, Jing Lei, Meng-Xi Fu, Qing Zhang, Wen Qin

**Affiliations:** 1 Institute of Food Processing and Safety, College of Food Science, Sichuan Agricultural University, Ya’an 625014, China; 2 Institute for Advanced Study, Chengdu University, Chengdu 610106, China

**Keywords:** *Dictyophora indusiata*, polysaccharides, in vitro digestion, SCFAs, gut microbiota

## Abstract

*Dictyophora indusiata* is one of the most famous edible mushrooms in China. *D. indusiata* polysaccharide (DP) has attracted increasing attention because of its multiple beneficial effects. In this study, the in vitro simulated digestion and microbial fermentation were designed to reveal the potential catabolic property of DP and its impacts on the modulation of gut microbial composition. The results showed that the reducing sugar content, total polysaccharides content, molecular weight, and rheological property of DP were not significantly altered under in vitro simulated digestive conditions. However, the molecular weight, apparent viscosity, and total polysaccharides content of indigestible DP (DPI) significantly decreased during in vitro fecal fermentation, and the reducing sugar content and the release of free monosaccharides notably increased, suggesting that DP could be degraded and used by gut microbiota. Additionally, the relative abundances of several beneficial bacteria, such as *Bacteroides*, *Catenibacterium*, *Parabacteroides*, and *Megamonas*, increased significantly, indicating that DP can regulate the composition and abundance of gut microbiota. Moreover, DP could also promote the production of SCFAs, thus changing the acid–base environment of the large intestine. The results of this study are beneficial for deeply clarifying the catabolic behavior of DP in the gastrointestinal tract, which can provide a theoretical basis for developing microbiota-directed products based on DP.

## 1. Introduction

*Dictyophora indusiata* belongs to the phylum *Basidiomycetes* and the family *Phallaceae*. *D. indusiata* is known as “the queen of mushrooms” due to its unusual and attractive appearance and its many health benefits [1]. In China, *D. indusiata* has a long history of cultivation, and it is favored by many people, not only for its rich taste, but also for its various health-promoting effects, such as antioxidant, immune regulation, anti-tumor, and neuroprotection effects [2,3,4,5]. Polysaccharides and their derivatives play an important role in life activities. According to previous studies, the carbohydrates in *D. indusiata* can reach 47% of the dry weight [1], and the polysaccharides from *D. indusiata* mainly contain a large amount of glucose and a small amount of galactose and mannose, mainly have a β-glucan structure, and exist as a triple helix conformation [3,4]. Because the human genome cannot produce enough carbohydrate active enzymes (CAZymes) to catabolize this kind of polysaccharide, the gut microbiota play a vital role in the catabolism of dietary polysaccharides [6,7].

Gut microbiota are a complex ecosystem of humans, and they can maintain the dynamic equilibrium in the host. Accumulating studies have recognized the critical roles of gut microbiota on the nutrition metabolism, host immunity, and other aspects of health [8,9]. When the gut microbiota are disordered, it may lead to obesity, diabetes, intestinal diseases, abnormal immune response, and other diseases [6]. Dietary polysaccharides can regulate the gut microbial community and be effectively utilized by specific gut microbiota to produce short-chain fatty acids (SCFAs) and other beneficial metabolic products [7,10]. It has been reported that the easily absorbed SCFAs can benefit the physiology of the host, such as enhancing energy absorption of intestinal epithelial cells, regulating host energy metabolism, and controlling body weight [10,11,12,13].

Recently, we have obtained a purified polysaccharide (DP) from the fruiting body of *D. indusiata*, which has been identified as a β-1,3-D-glucan with β-1,6-D-Glc*p* as branched chains. DP has a high molecular weight of 1.072 × 10^6^ Da and exhibits a rigid-rod chain conformation in NaCl solution. Additionally, DP possesses remarkable immunostimulatory activity in vivo, which is closely associated with its molecular weight and chain conformation [14]. In fact, the bioactivities of dietary polysaccharides are closely associated with their catabolism from the stomach to the colon [15]. Nevertheless, the digestive behavior and microbial fermentation characteristic of DP, as well as its potential to modulate the microbial composition, are still unclear. Therefore, in this study, the in vitro simulated digestion and microbial fermentation were designed to elucidate the potential catabolic property of DP in the gastrointestinal tract, which could lay a theoretical foundation for the development of microbiota-directed products based on DP.

## 2. Materials and Methods

### 2.1. Materials and Chemicals

The fruiting bodies of *D. indusiata* were purchased from Yibin, Sichuan Province, China. The raw materials were dried at 45 °C and milled into powders, then stored at −20 °C for further analysis. α-Amylase (1000 U/mg), pancreatin (4000 U/g), pepsin (3000 U/g), bile salt, cysteine, hemin, 1-phenyl-3-methyl-5-pyrazolone (PMP), resazurin, and vitamin K1 were all bought from Sigma-Aldrich (St. Louis, MO, USA). Other chemicals and reagents used in this work were of analytical grade.

### 2.2. Extraction and Isolation of Polysaccharides

The extraction and isolation of *D. indusiata* polysaccharide (DP) were carried out according to our prior methods [14]. In brief, DP was extracted by adding *D. indusiata* powder (1.0 g) into 22.0 mL of deionized water with the ultrasonic extraction amplitude of 57% and ultrasonic extraction time of 15 min. After ultrasound-assisted extraction, 95% of ethanol (*v*/*v*) was added in the supernatant at a volume ratio of 3:1 and settled for 12 h. After the precipitation was redissolved, the impurities were further removed by continuous precipitation with 95% (*v*/*v*) of ethanol at a final concentration of 30% (*v*/*v*). Afterwards, the precipitate was re-dissolved and then ultra-filtered with a 100.0 kDa ultrafiltration membrane. Finally, DP was obtained via freeze-drying at −80 °C for 48 h.

### 2.3. In vitro Simulated Digestion and Microbial Fermentation of DP

#### 2.3.1. In Vitro Simulated Digestion Procedure

**Figure 1:**
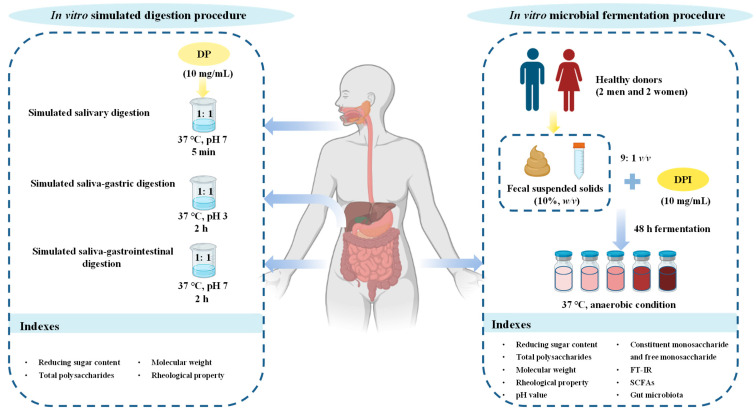
A schematic of the experimental designs of in vitro simulated digestion and microbial fermentation.

A schematic of the experimental designs of in vitro simulated digestion and microbial fermentation is shown in Figure 1. In brief, the preparation of simulated saliva, gastric juice, and intestinal juice in vitro refers to the method of Brodkorb et al. [16]. The simulated salivary stock solution (0.4 L) was composed of 0.5 M KCl (30.2 mL), 0.5 M KH_2_PO_4_ (7.4 mL), 1 M NaHCO_3_ (13.6 mL), 0.15 M MgCl_2_(H_2_O)_6_ (1.0 mL), 0.5 M (NH_4_)_2_CO_3_ (0.12 mL), and 6 M HCl (0.18 mL). A total of 10 mg/mL of DP solution was mixed with simulated salivary stock solution (1:0.8, *v*/*v*), and then α-amylase was added. The final concentration of α-amylase in the system was 75 U/mL, and the final concentration of CaCl_2_ (H_2_O)_2_ in the system was 1.5 mM. After fully mixing, the sterile water was added to adjust the final volume ratio of the DP solution and simulated saliva into 1:1 (*v*/*v*), and the mixture was reacted at 37 °C. After reaction for 0, 2, and 5 min, respectively, 10 mL of reaction solution was taken out and quickly placed in boiled water for 5 min.

The simulated gastric stock solution (0.4 L) was composed of 0.5 M KCl (13.8 mL), 0.5 M KH_2_PO_4_ (1.6 mL), 2 M NaCl (23.6 mL), 1 M NaHCO_3_ (25 mL), 0.15 M MgCl_2_(H_2_O)_6_ (0.8 mL), 0.5 M (NH_4_)_2_CO_3_ (1.0 mL), and 6 M HCl (2.6 mL). The residual reaction solution of salivary digestion was mixed with the simulated gastric stock solution (1:0.8, *v/v*), and pepsin and 0.3 M CaCl_2_ (H_2_O)_2_ were added to make the final concentration of 2000 U/mL and 0.15 mM in the system, respectively. In addition, the pH value of the system was adjusted to 3. After fully mixing, the sterile water was added to adjust the final volume ratio of the residual reaction solution of salivary digestion and the simulated gastric juice into 1:1 (*v*/*v*); it was then reacted at 37 °C. After reaction for 0, 0.5, 1, and 2 h, respectively, 10 mL of reaction solution was also taken out and quickly placed in boiled water for 5 min.

The simulated intestinal stock solution (0.4 L) was composed of 0.5 M KCl (13.6 mL), 0.5 M KH_2_PO_4_ (1.6 mL), 2 M NaCl (19.2 mL), 1 M NaHCO_3_ (85 mL), 0.15 M MgCl_2_(H_2_O)_6_ (2.2 mL), and 6 M HCl (1.4 mL). The residual reaction solution of gastric digestion was blended with the simulated intestinal stock solution (1:0.8, *v*/*v*), and 0.3 M CaCl_2_ (H_2_O)_2_, pancreatin, and bile salt were added to make the final concentrations in the system of 0.6 mM, 20 U/mL, and 10 mM, respectively. Then, the pH value of the system was adjusted to 7. After fully mixing, the sterile water was also added to adjust the final volume ratio of the residual reaction solution and the simulated intestinal solution into 1:1 (*v*/*v*), and the reaction was conducted at 37 °C. After reaction for 0, 0.5, 1, and 2 h, respectively, 10 mL of reaction solution was also taken out and quickly placed in boiled water for 5 min.

The digested solution was centrifuged and utilized for the determination of reducing the sugar content, and the rest of the solution was used to detect the physicochemical properties. Different digestive samples were named as DP-O (digested by oral digestion), DP g (digested by oral–gastric digestion), and DP-I (digested by oral–gastrointestinal digestion), respectively.

#### 2.3.2. In Vitro Simulated Fermentation Procedure

As shown in Figure 1, the simulated fermentation of indigestible *D. indusiata* polysaccharide (DPI) in vitro was also conducted via the former method with minor modifications [10]. Fresh feces were collected from four healthy donors (2 men and 2 women, aged from 18–25) who were required to eat a normal diet (Chinese diet). A minimum of 3 months of treatment with antibiotics or probiotics was forbidden to donors. The stool donors all started defecating at the same time, and immediately after defecation, samples were taken and mixed evenly to prepare stool homogenates. Equal quantities of donors’ feces were mixed and then diluted with asepsis ameliorative normal saline (cysteine-HCl 0.5 g/L, NaCl 9.0 g/L) to prepare fecal suspended solids (10%, *w*/*v*). The fermentation medium (1 L) was composed of 2.0 g of yeast extract, 2.0 g of peptone, 0.1 g of NaCl, 0.04 g of K_2_HPO_4_, 0.04 g of KH_2_PO_4_, 0.01 g of CaCl_2_ · 6H_2_O, 0.01 g of MgSO_4_ · 7H_2_O, 2.0 g of NaHCO_3_, 0.02 g of heme, 0.5 g of cysteine hydrochloride, 0.5 g of bile salt, 2.0 mL of Tween 80, 1.0 mg of rezamycin, and 10 µL of vitamin K. 

The fecal-suspended solid was added into the fermentation medium containing DPI (10 mg/mL, DPI group), the fermentation medium without other carbon sources (CK group), and the normal saline (Feces group) to make their volume ratio into 1:9. Afterwards, the mixture was fermented in an anaerobic incubator (BPN-300CS, Being Instrument, Shanghai, China) at 37 °C. At 0, 6, 12, 24 and 48 h fermentation, the starter fermentation solution was taken for detection and analysis. The samples obtained at different fermentation time points were named as DPI-0 h, DPI-6 h, DPI-12 h, DPI-24 h, and DPI-48 h, respectively. The gut microbial composition of the fermentation broth after in vitro fermentation for 48 h was analyzed.

### 2.4. Determination of Physicochemical Properties of DP during In Vitro Digestion and Fermentation

#### 2.4.1. Determination of pH Value, Reducing Sugar Content (C_R_), and Total Polysaccharides Content

The pH value of the fermentation broth at each time point was measured by using a pH Meter (RMD-H800, Shanghai Lanchang Automation Technology Co., Ltd., Shanghai, China). The C_R_ was detected using the DNS method with glucose as a reference. The evaluation of total polysaccharides content of DP, its digested samples (DP-O, DP-G, and DP-I), and its fermented samples (DPI-0 h, DPI-6 h, DPI-12 h, DPI-24 h, and DPI-48 h) was based on the phenol–sulfuric acid method with glucose as a reference. The total carbohydrate consumption was calculated to reflect the fermentability of DPI.

#### 2.4.2. Determination of Molecular Weight

High-performance size-exclusion chromatography (HPSEC) was used to determine the molecular weights (*M_w_*) and polydispersities (*M_w_*/*M_n_*) of DP, its digested samples (DP-O, DP-G and DP-I), and its fermented samples (DPI-0 h, DPI-6 h, DPI-12 h, DPI-24 h, and DPI-48 h) with a multi-angle laser light scattering and a refractive index detector (MALLS-RID, Wyatt Technology Co., Santa Barbara, CA, USA) [17]. The samples were separated using the Shodex OHpak SB-806 M HQ column (300 mm × 8.0 mm, i.d., Shodex, Japan) at a flow rate of 0.5 mL/min in a mobile phase of 0.9% (*w*/*v*) NaCl.

#### 2.4.3. Determination of Rheological Property

According to the formerly reported method, a Discovery Hybrid Rheometer-1 (DHR-1, TA instruments, New Castle, DE, USA) was applied for rheological characterization of DP, its digested samples (DP-O, DP g and DP-I), and its fermented samples (DPI-0 h, DPI-6 h, DPI-12 h, DPI-24 h, and DPI-48 h) at a concentration of 20 mg/mL [10].

#### 2.4.4. Determination of Constituent Monosaccharide and Released Free Monosaccharide

Based on the previous method, high-performance liquid chromatography (HPLC, Thermo Fisher Scientific U3000, Waltham, MA, USA) was applied for the constituent monosaccharide and released free monosaccharide determination [7]. The phenomenex gemini 5u C18 110A column (150 mm × 4.6 mm, 5 μm) was utilized. The phosphate-buffered solution (0.1 M, pH = 6.7) mixed with acetonitrile at a volume ratio of 83:17 was applied as the mobile phase. The mobile phase was eluted at the rate of 1.0 mL/min and measured at a 245-nm detection wavelength.

#### 2.4.5. Fourier Transform Infrared (FT-IR) Spectroscopy Analysis

According to our previous method [10], the FT-IR characteristic bands of fermented samples (DPI-0 h, DPI-6 h, DPI-12 h, DPI-24 h, and DPI-48 h) were measured by using a Nicolet iS 10 FT-IR (Thermo Fisher Scientific, Waltham, MA, USA) in the frequency range of 500–4000 cm^−1^.

#### 2.4.6. Determination of SCFAs

According to our previous study, the concentration of SCFAs produced during different fermentation stages was determined by using gas chromatography (GC, Agilent 7890B GC system (Agilent Technologies, Santa Clara, CA, USA) [10]. In brief, the fermented samples were centrifuged (6000× *g*, 10 min), and then the supernatant was sucked and mixed with an equal volume of internal standard solution (0.05 M 2-ethylbutyric acid). After filtration using 0.22 μm of the organic filter membrane, 1 μL of the sample was injected and separated using the HPINNOWAX column (30 m × 0.25 cm × 0.25 μm, Agilent Technologies, Palo Alto, CA, USA). Likewise, a calibration curve of the standards (acetic acid, propionic acid, *n*-butyric acid, *i*-butyric acid, *n*-valeric acid, and *i*-valeric acid) was established to calculate the concentration of SCFAs in each sample.

#### 2.4.7. Analysis of Gut Microbiota

DNA was extracted from the samples after fermentation for 48 h, and then primers were designed based on the conserved region, which was performed as previously reported [18]. Briefly, DNA in fermented mixtures was extracted by using a QIAamp DNA stool mini kit (Qiagen, Shanghai, China) according to the manufacturer’s procedures. Next, universal primers (338F and 806R) were applied for the amplification of the 16S rRNA gene. The targeted sequences were amplified via PCR and its products were purified, quantified, and homogenized to get a sequencing library. On this basis, the library QC was performed for constructing libraries. Qualified libraries were sequenced using Illumina Novaseq 6000. First, the original sequenced sequence was filtered via Trimmatic v 0.33. Then, the primer sequence was identified and eliminated using cutadapt 1.9.1 to get the high-quality reads. Based on overlapping sequences, the filtered sequence of each sample was spliced using Usearch v.10.0, and then the length of the spliced data was filtered according to the length range of different regions. Chimeric sequences were discriminated and eliminated via UCHIME v4.2, and the effective data were obtained. Reads with similarity above 97.0% were clustered using Usearch to obtain OTUs. SILVA was used as the reference database to count the composition of the sample communities at different levels, and we applied QIIME software to create species abundance charts at different classification levels. Gene functions were predicted via PICRUSt software and analyzed according to the Kyoto Encyclopedia of Genes and Genomes (KEGG) database.

### 2.5. Statistical Analysis

All experiments were expressed as mean ± standard deviations of triplicate determinations. Statistical analysis was performed by OriginLab 2022 (OriginLab Corporation, Northampton, MA, USA). One-way analysis of variance (ANOVA) with Duncan’s multiple range test was carried out to determine any significant differences (*p* < 0.05).

## 3. Results and Discussion

### 3.1. Digestive Properties of DP during In Vitro Simulated Digestion

#### 3.1.1. Reducing Sugar Content

During the initial digestion process, the C_R_ in DP was 0.127 ± 0.011 mg/mL (Table 1). At the end of the salivary digestion stage, the C_R_ had raised to 0.129 ± 0.002 mg/mL, indicating that DP could not be decomposed during oral digestion. This result may be due to the fact that, as the main enzyme in human saliva, salivary α-amylase usually acts on α-1,4-glycosidic linkages [19]. Similarly, the C_R_ also had no significant change during gastric digestion, ranging from 0.252 ± 0.054 mg/mL to 0.295 ± 0.011 mg/mL. The presence of substances such as an acidic environment, electrolytes, and digestive enzymes in the stomach may have caused the change in the C_R_ from the oral phase to the gastric phase. However, there was no significant change in the C_R_ in the gastric phase, indicating that DP was not broken down and destroyed. Similar results were also found in the gastric digestion of *Gracilaria rubra* polysaccharides [20]. Due to the presence of reducing sugar in the simulated small intestine fluid, the C_R_ in the initial simulated small intestine digestion increased to 0.789 ± 0.137 mg/mL [20]. However, the C_R_ did not change significantly during the small intestinal digestion, indicating that DP was still stable at this digestion stage. In addition, the total polysaccharides content in DP, DP-O, DP-G, and DP-I was also stable, being in the range of 95.33%–96.53%. These results indicated that DP was overall stable under in vitro simulated digestive conditions, which might be attributed to the fact that DP has a high molecular weight of 1.072 × 10^6^ Da and exhibits a rigid-rod chain conformation in NaCl solution [14].

#### 3.1.2. Molecular Weight

**Figure 2:**
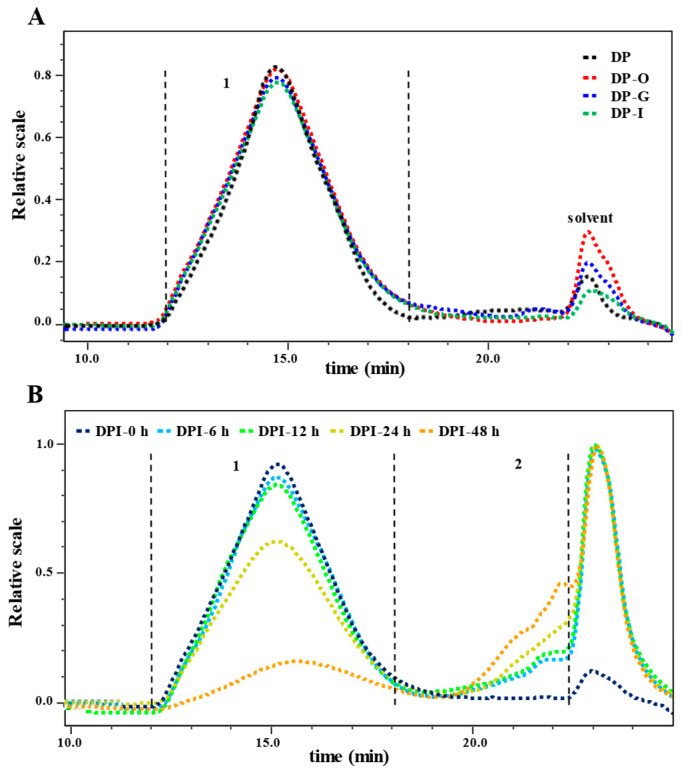
Changes in size exclusion chromatograms of *D. indusiata* polysaccharide during in vitro digestion (**A**) and microbial fermentation (**B**). The sample codes were consistent with that in Table 1.

As shown in Table 2, the molecular weights of DP, DP-O, DP-G, and DP-I were 1.072 × 10^6^, 1.066 × 10^6^, 1.063 × 10^6^, and 1.061 × 10^6^ Da, respectively. As shown in Figure 2A, it could be seen that the peak did not move during in vitro simulated digestion, which meant that DP could not be digested. This result was analogous to the experimental results of previous studies [21,22]. Results further confirmed that DP was overall stable under in vitro simulated digestive conditions.

#### 3.1.3. Rheological Properties

**Figure 3:**
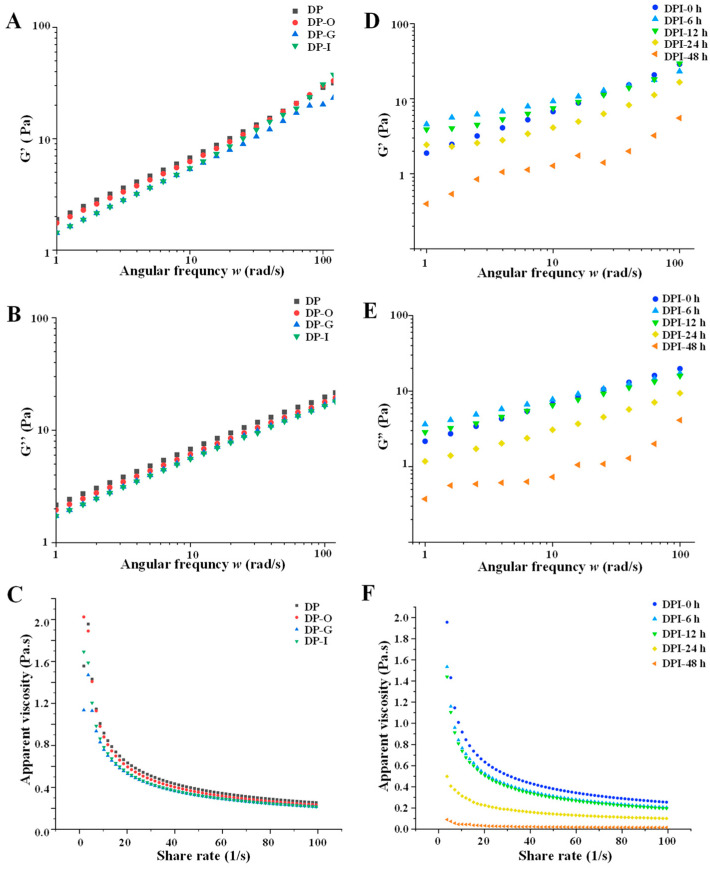
Changes in storage modulus, loss modulus, and apparent viscosity of *D. indusiata* polysaccharide during in vitro digestion (**A**–**C**) and microbial fermentation (**D**–**F**). The sample codes were consistent with that in Table 1.

At 25 °C, the flow behaviors of DP before and after simulated digestion were determined over a shear rate range of 1–100 s^−1^. The results showed that the apparent viscosities of the samples (DP, DP-O, DP-G, and DP-I) were affected by the shear rate, respectively, and showed shear thinning behavior and Newtonian fluid behavior (Figure 3C). At the low shear rate, the typical shear thinning behavior could be observed, and there was a negative correlation between apparent viscosity and shear rate. Then, at the high shear rate, the samples showed a Newtonian plateau and remained at the apparent viscosity almost unchanged. This result was consistent with our previous results [10,18]. As shown in Figure 3A,B, the storage modulus (G′) of the samples at the low frequency was higher than the loss modulus (G″), which meant the viscoelastic behaviors of the samples were typical [23]. However, the opposite results were observed at high frequency, indicating that the samples had obvious trends in liquid properties, respectively. These results suggested that in vitro simulated digestion did not affect the G″ and G′ of DP. Overall, the apparent viscosity and modulus results indicated that DP was overall stable across different simulated digestion stages.

### 3.2. Microbial Fermentation Characteristic of DPI during In Vitro Fecal Fermentation

#### 3.2.1. Changes in Total Polysaccharides Content and Reducing Sugar Content

According to the above results, the simulated digestive solutions did not break down DP, and the indigestible DP (DPI) could reach the large colon almost completely. As shown in Table 1, during the process of in vitro microbial fermentation, the C_R_ of DPI did not change significantly at the time points from 0 h to 12 h, but increased sharply during 12–24 h (0.179 ± 0.005 to 1.539 ± 0.002 mg/mL) and continued to increase until 48 h (2.585 ± 0.021 mg/mL). This result was due to the fact that enzymes released by gut microbiota can act on most non-digestive polysaccharides, decompose the glycosidic linkages of polysaccharides, and produce reducing sugars, thereby stimulating the growth of gut microbiota, which leads to a reduction in the total polysaccharides content [7,10,24]. As shown in Table 2, after the microbial fermentation for 48 h, the total polysaccharides content of DPI decreased from 95.01 ± 1.07% to 51.12 ± 1.01%. At the end of microbial fermentation, the fermentation rate of DPI gradually increased to 46.20%, further indicating that DPI was continuously utilized and decomposed by gut microbiota. Previous studies have shown that intestinal microorganisms, especially *Bacteroides*, can use dietary polysaccharides as carbon sources for growth [7,20,25].

#### 3.2.2. Changes in Molecular Weight

As shown in Table 2, the molecular weight of fraction 1 in DPI gradually decreased from 1.057 × 10^6^ to 0.595 × 10^6^ Da during the fermentation. Particularly, as demonstrated in Figure 2B, the retention time and peak area of DPI barely altered during the fermentation from 0 h to 12 h. However, after fermentation for 24 h, a degraded fragment (fraction 2) was found in DPI-24 h, and the peak area of fraction 1 in DPI-24 h significantly decreased. Indeed, the molecular weight of fraction 1 in DPI-24 h significantly decreased to 0.851 × 10^6^ Da, and its peak area decreased to 81.1%, while the molecular weight and peak area of fraction 2 increased to 1.519 × 10^5^ Da and 18.9%, respectively, indicating that the gut microbiota significantly decomposed DPI during the fermentation from 12 h to 24 h. Additionally, as the fermentation time progressed from 24 h to 48 h, the molecular weight of fraction 1 in DPI decreased from 0.851 × 10^6^ to 0.595 × 10^6^ Da, demonstrating that DPI was broken down into fragments by microorganisms for their growth. Similar phenomena have been observed in the experimental results of other researchers [7,10,20,25]. 

#### 3.2.3. Changes in Monosaccharide Composition and Released Free Monosaccharide

**Figure 4:**
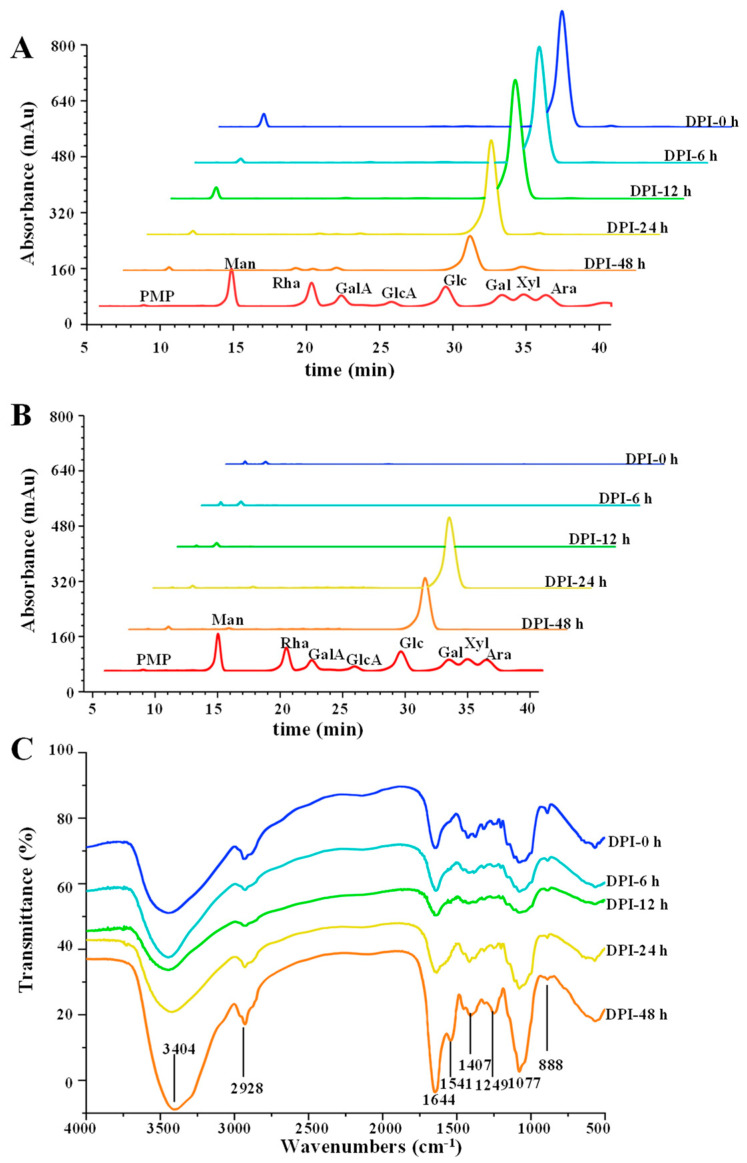
Changes in constituent monosaccharide (**A**), released free monosaccharide (**B**), and FT-IR spectra (**C**) of DPI during in vitro fermentation. PMP, 1-phenyl-3-methyl-5-pyrazolone; Man, mannose; Rha, rhamnose; GlcA, glucuronic acid; GalA, galacturonic acid; Glc, glucose; Gal, galactose; Xyl, xylose; Ara, arabinose.

As shown in Figure 4A, the contents of glucose in DPI-0 h, DPI-6 h, and DPI-12 h did not change significantly, but the contents of glucose in DPI-24 h and DPI-48 h decreased significantly, which were in agreement with the changes in total polysaccharides content and C_R_. The released free glucose was not measured in the fermentation broth of DPI-0 h, DPI-6 h, and DPI-12 h (Figure 4B), indicating that the gut microbiota were unable to effectively degrade DPI into monosaccharides during the fermentation from 0 h to 12 h [20]. The released free glucose was detected in the fermentation broth of DPI-24 h, which may be due to the combined action of microbial degradation and microbial utilization [7,20]. The decrease in released free glucose after the fermentation for 48 h indicated that the utilization rate of free glucose by gut microbiota was higher than the decomposition rate of DPI by gut microbiota, resulting in the decrease in glucose content [7,26].

#### 3.2.4. Changes in FT-IR Spectra

As shown in Figure 4C, during the fermentation process, the characteristic absorption bands of the FT-IR spectra of the fermented samples were analogous. More specifically, the O-H tensile vibration had a connection with the absorption peak at 3404 cm^−1^ [27]. The peak at 2928 cm^−1^ was due to the C-H stretching vibration of the methylene group [28]. The signal peak of 1644 cm^−1^ may be due to bound water, and the C-O contraction vibration corresponded to the signal at 1407 cm^−1^ [4]. The absorption peak at 1250 cm^−1^ was according to C-O-C [25]. The signal at 1077 cm^−1^ came from the pyranosyl ring [29]. Signal representation at 888 cm^−1^ was attributed to β-glucan [30]. In addition, the infrared spectrum of DPI-48 h showed a small signal peak at 1541 cm^−1^, which may be N-H bending vibration or C-N stretching vibration of amide band, indicating that several bacteria could produce protein metabolites [29]. 

#### 3.2.5. Changes in Rheological Properties

The apparent viscosity of polysaccharides perhaps changes the growth of microorganisms by influencing the permeation rate of nutrients, water, and oxygen in the medium [10,31]. Figure 3F showed the changes in apparent viscosity of DPI during in vitro fermentation. The results showed that the fermented samples showed non-Newtonian shear thinning behavior in the 0.01–50 s^−1^ shear rate range, but showed near Newtonian flow behavior in the 50–100 s^−1^ shear rate range [32]. Compared with DPI-0 h, DPI-6 h, and DPI-12 h, the apparent viscosity of DPI-24 h and DPI-48 h decreased significantly. This result may be due to the reduction in the molecular weight of DPI via simulated in vitro fermentation, which led to the decrease in viscosity and the change in the flow behavior of the polysaccharides solution, further supporting that human gut microbiota could utilize DPI [23]. During the whole fermentation process, the storage modulus (G′) and the loss modulus (G″) of the fermented samples decreased over time, and DPI-24 h and DPI-48 h decreased significantly (Figure 3D,E). This result may be related to the decrease in the total polysaccharides content of the fermented samples. In previous studies, it was found that the concentration of polysaccharides was closely associated with the magnitude of the loss modulus (G″) [33].

#### 3.2.6. Changes in pH Value and Fermentation Products

**Figure 5:**
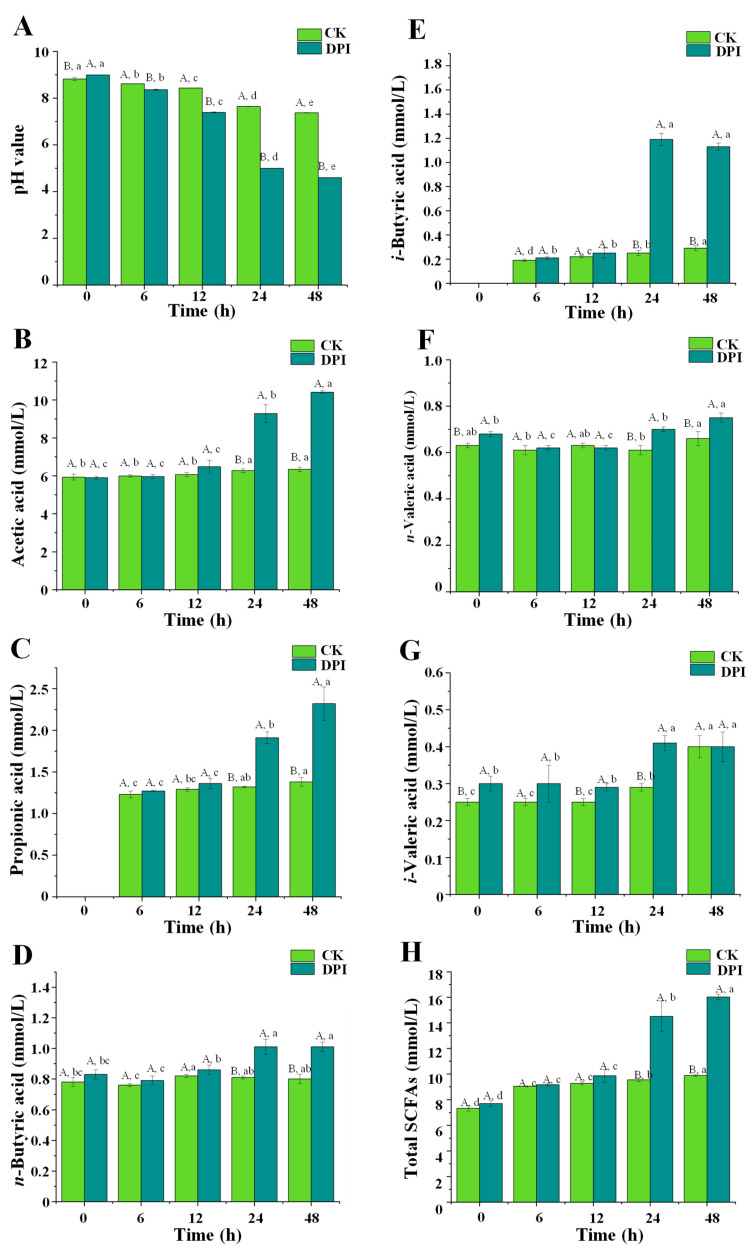
Changes in pH value (**A**), acetic acid (**B**), propionic acid (**C**), *n*-butyric acid (**D**), *i*-butyric acid (**E**), *n*-valeric acid (**F**), *i*-valeric acid (**G**), and total SCFAs (**H**) of DPI during in vitro fermentation. CK, the negative control group; DPI, the DPI group; different capital letters represent significant differences (*p* < 0.05) at the same time in different groups (A–B). Different lowercase letters represent significant differences (*p* < 0.05) in the same group at different times (a–e).

Compared with the CK group, the pH value of DPI decreased significantly (Figure 5A), which decreased from 8.99 to 4.60, while the CK group only decreased from 8.82 to 7.37. It was probably due to the fact that DPI could promote the microbial metabolism to generate SCFAs [25]. This result was similar to the results of changes in the pH value and SCFAs of *Cookeina speciosa* glucans during in vitro fermentation, which also had a large molecular weight (2 × 10^6^ Da) [34]. More specifically, the pH value slightly decreased at the initial fermentation stage from 0 h to 12 h in the DPI group, while it decreased sharply from 12 h to 48 h. The low pH caused by SCFA production can promote some positive physiological effects in the colon. It was found that the low pH was considered to reduce the efficiency of conversion of primary bile acids to secondary bile acids and reduce their carcinogenic potential [35]. It was worth noting that the change behavior of pH value was almost consistent with the dynamic changes in total polysaccharides content and molecular weight in the DPI group during in vitro microbial fermentation. Several studies have shown that SCFAs can regulate energy expenditure and effectively control body weight as well as regulate epithelial cell proliferation and maintain epithelial barrier function to prevent colorectal cancer [11,12,36]. During the microbial fermentation from 12 h to 48 h, the content of total SCFAs in the DPI group increased significantly from 7.7 ± 0.22 mmol/L to 16.02 ± 0.22 mmol/L (Figure 5H). This result showed that the fecal microbial activity was lower in the early stage of fermentation (0–12 h) and higher in the middle (12–24 h) and late (24–48 h) stages of fermentation, which had a bearing on the change in pH value. Similar fermentation characteristics have also appeared in previous reports [29,37]. 

It can be seen from Figure 5B–G that the contents of acetic acid, propionic acid, *n*-butyric acid, *i*-butyric acid, *n*-valeric acid, and *i*-valeric acid increased significantly in the DPI group after in vitro fermentation for 48 h when compared with those of the CK group (*p* < 0.05). Indeed, these SCFAs increased rapidly during the fermentation from 12 h to 48 h, which was consistent with the dynamic changes in other physicochemical properties of DPI. The main incremental contributions of SCFAs, including acetate acid, propionic acid, and *i*-butyric acid, increased to 10.41 ± 0.77 mmol/L, 2.32 ± 0.20 mmol/L, and 1.13 ± 0.03 mmol/L, respectively. This result was also similar to previous studies [18,24]. Acetic acid can improve glucose homeostasis and insulin excretion in animal studies and has good regulation effects on liver function and cholesterol metabolism function [25,38]. Propionic acid, produced in the colon, may have a direct useful effect on adipose tissue by lessening obesity-associated inflammation and promoting glucose uptake and lipogenesis [39]. As an important energy substrate for maintaining the activity of colonic epithelial cells, butyrate has anti-inflammatory effects, and can also inhibit obesity and antagonize tumor production [21,25]. Acetic acid and propionic acid may be produced by *Bacteroidetes* through a series of glycoside hydrolases and carbohydrate metabolic pathways [21,40]. *Clostridium* contributes to the production of butyrate, and *Megamonas* also contributes to the production of SCFAs [41]. Although *n*-valeric acid and *i*-valeric acid increased significantly after in vitro fermentation for 48 h, their levels were low, perhaps owing to the fact that the microorganisms producing these SCFAs constituted only a small fraction of gut microbiota [37]. The small amount of SCFAs produced in the CK group after in vitro fermentation for 48 h may be due to the decomposition of some branched chain amino acids such as valine, leucine, and isoleucine [40].

#### 3.2.7. Effect of DPI on the Microbial Composition

##### Diversity Analysis

**Figure 6:**
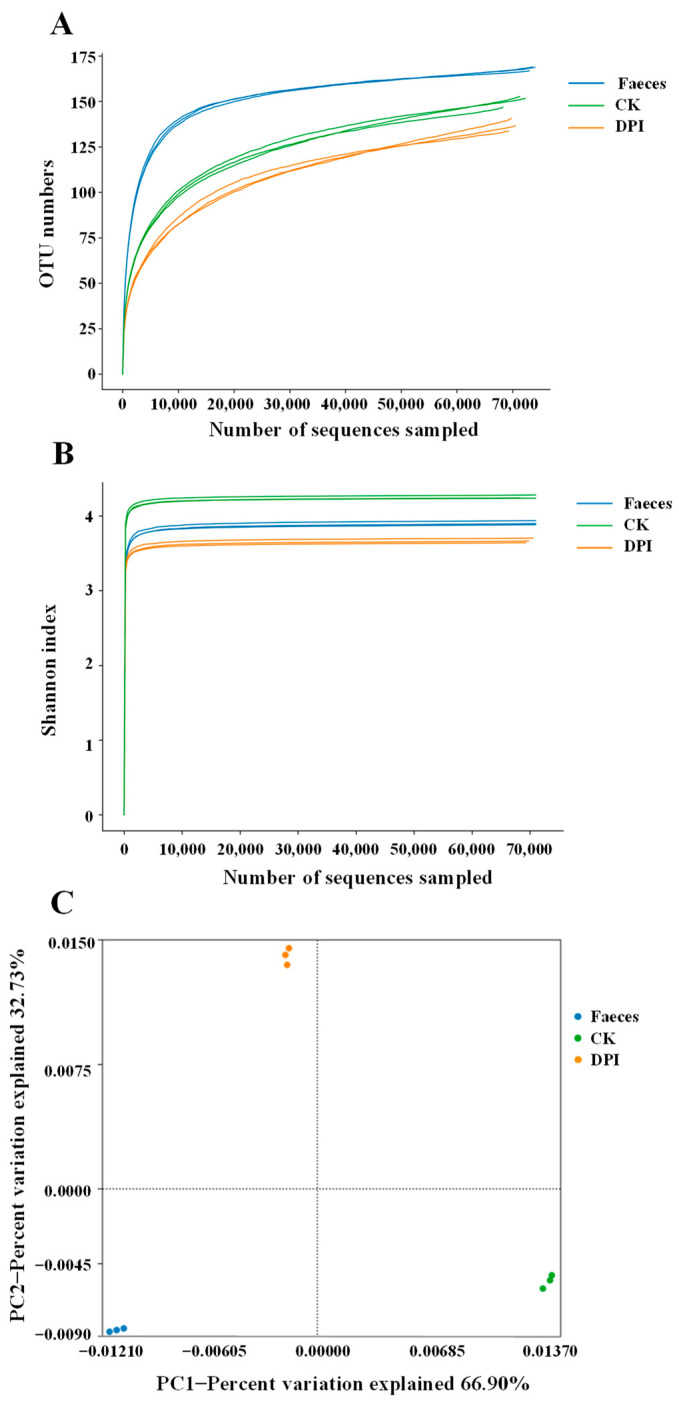
Dilution curve (**A**), Shannon index (**B**), and PCA (**C**) of the gut microbiota of the different fermented samples. Feces, the original feces group; CK, the negative control group; DPI, the DPI group.

The dilution curve and Shannon index are important indexes in α-diversity analysis, reflecting the species abundance and species diversity of samples. As shown in Figure 6A, when the sequencing depth and sample size were enlarged, it was difficult to find more OTUs, which suggested that the sample size and sequencing depth were sufficient to investigate gut microbiota. Similarly, the larger the Shannon index, the more species. When the curve tends to be gentle, the quantity of the sequencing data was sufficiently large (Figure 6B). It can be seen from both images that the abundance and diversity of the OTUs of the gut microbiota in the DPI group were significantly lower than those in the CK and Feces groups. The decrease in the Shannon index might be attributed to the competitive effect of the dominant microbial community, which is a common phenomenon after simulated fermentation of dietary polysaccharides with intestinal regulation function [25]. Principal component analysis (PCA) can show the genetic relationship of microbial communities among different groups [21]. As shown in Figure 6C, PC1 and PC2 contributed for 66.90% and 32.73% of the variation, respectively, which meant that there was a significant discrepancy in the gut microbial composition among DPI, CK, and Feces groups. The overall results showed that the gut microbial composition of the DPI group changed significantly compared with the CK and Feces groups. 

##### Species Annotation and Taxonomic Analysis

**Figure 7:**
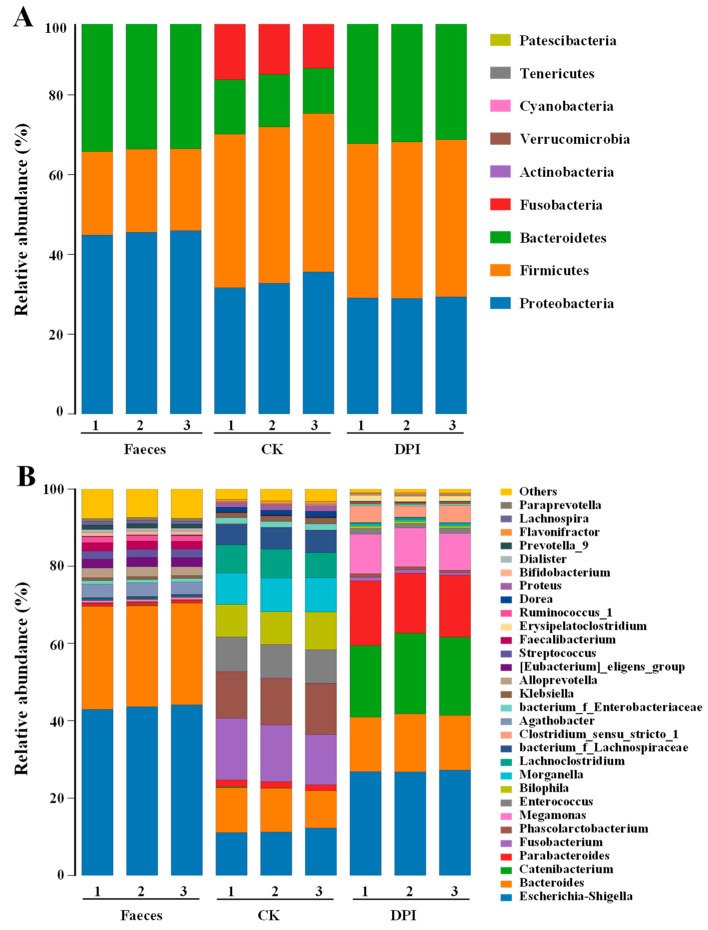
Species distribution at the phylum (**A**) and genus (**B**) levels of the different fermented samples. Feces, the original feces group; CK, the negative control group; DPI, the DPI group.

At the phylum level (Figure 7A), the DPI group was mainly made up of 39.10% *Firmicutes*, 30.54% *Bacteroidetes*, and *29.15% Proteobacteria*, and the remaining 1.21% was mainly composed of *Fusobacteria*, *Actinobacteria,* and others. The dominant bacteria in the CK group were identified as 39.12% *Firmicutes*, 33.38% *Proteobacteria*, 14.49% *Fusobacteria,* and 12.71% *Bacteroidetes*. The Feces group was mainly composed of 45.49% *Proteobacteria*, 20.74% *Firmicutes*, and 32.48% *Bacteroidetes*. Compared with the CK and Feces groups, the DPI group significantly improved the relative abundance of *Bacteroidetes* and significantly reduced the relative abundances of *Proteobacteria* and *Fusobacteria*, which indicated that DPI had beneficial effects on the health of gut microbiota, which was similar to the effects of polysaccharides from *Lentinula edodes* and *Pleurotus eryngii* on regulating gut microbiota [26,42]. Recently, it has been found that the reduction of *Firmicutes* and *Bacteroidetes* and the imbalance of excessive growth of *Proteobacteria* and *Bacillus* are the characteristics of patients with inflammatory bowel disease (IBD) [43]. It can be seen that DPI has a positive, promoting effect on IBD. In addition, *Bacteroidetes* is one of the major gut microorganisms in charge of polysaccharide breakdown [44]. The expression of polysaccharide-degrading enzymes increases in *Bacteroidetes* and decreases in *Firmicutes* [45]. One of the biological indicators of obesity is the ratio of *Firmicutes*-to-*Bacteroidetes* (F/B), which is usually higher in obese patients [44]. DPI decreased the F:B ratio by raising the abundance of *Bacteroidetes*, which indicated that DPI might have the effect of reducing obesity. This result was similar to the results found in relation to polysaccharides from loquat leaves, okra, and *Gracilaria rubra* [7,20,24]. *Fusobacteria* are generally considered to be pathogens that increase conditionality due to nutritional lack [44]. The relative abundance of *Proteobacteria* is closely related to the function of intestinal epithelial cells, intestinal inflammation, and immune imbalance, which is an important index to measure intestinal health [46]. Collectively, these results indicated that DPI was able to regulate the composition of intestinal microorganisms, promoting the growth of several beneficial bacteria in particular.

At the genus level (Figure 7B), the DPI group was principally made up of 27.03% *Escherichia-Shigella*, 14.3% *Bacteroides*, 19.90% *Catenibacterium*, 16.14% *Parabacteroides*, 9.99% *Megamonas*, 3.90% *Clostridium_sensu_stricto_1*, and 1.45% *Erysipelatoclostridium*. Compared with the CK and Feces groups, the DPI group significantly increased the abundances of *Catenibacterium*, *Parabacteroides*, *Megamonas,* and *Clostridium_sensu_stricto_1*. *Catenibacterium* is able to metabolize acetic acid, butyric acid, and *i*-butyric acid using glucose, as extracted from oats β-glucan [47,48]. Recently, the in vivo study has found that *Parabacteroides* can reduce obesity, insulin tolerance, lipid metabolism chaos, and nonalcoholic fatty liver symptoms [49], and it can secrete β-glucosidase to participate in body metabolism and regulation and maintain the symbiotic relationship between mammals and microorganisms [50]. Based on the analysis of gut microbial composition in patients with cerebral infarction and ischemia, it was observed that the disease results in a low relative abundance of *Megamonas* in patients [51]. Therefore, DPI can increase *Megamonas* abundance, which was beneficial to reversing regulation of cerebral infarction and ischemia. Recently, *Clostridium* was proved to promote the induction of colonic Treg. TLR9 signaling can maintain immune homeostasis by limiting the transformation of Treg cells in the intestinal tract [52,53]. 

The DPI group also inhibited the proliferation of deleterious pathogenic bacteria compared with the CK and Feces groups. *Escherichia-Shigella, Fusobacterium, Bilophila, Morganella*, and *Lachnoclostridium* are all harmful pathogens in intestinal microorganisms. *Fusobacterium* is related to the pathogenesis of gastric cancer [54]. *Bilophila* can aggravate metabolic dysfunction induced by a high-fat diet in mice [55]. There is a clear relationship between *Morganella* and depression. Increasing its abundance will make the risk of depression higher than that of others [56]. *Lachnoclostridium* can cause atherosclerosis by increasing trimethylamine oxide damage to aortic health [57]. The relative abundance of *Escherichia-Shigella* increased in the DPI group. The reason for this phenomenon might be in connection with the growth of *Escherichia-Shigella* by using low molecular weight carbon sources to support itself [37].

**Figure 8:**
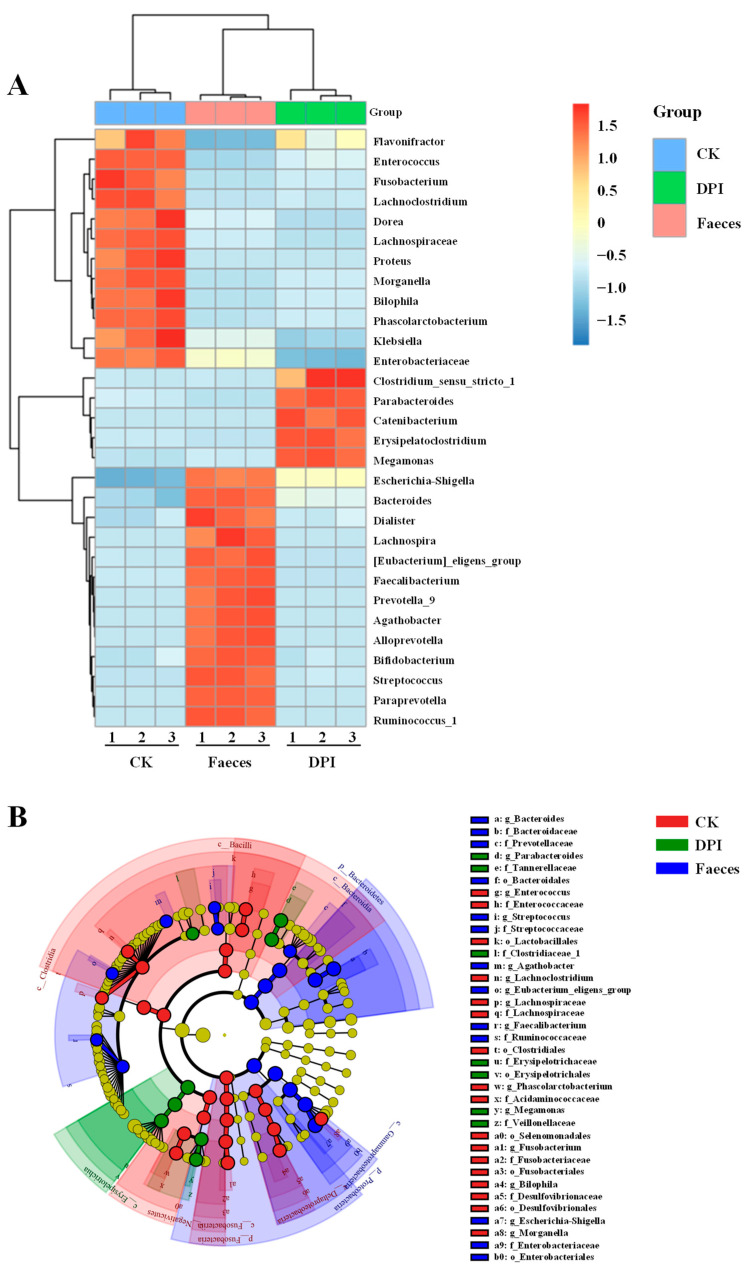
Heatmap analysis at the genus level (**A**) and Cladogram based on LEfSe analysis (**B**) of gut microbiota for different fermented samples. Feces, the original feces group; CK, the negative control group; DPI, the DPI group.

The heat map of microbial composition is shown in Figure 8A, showing the abundance of the first 30 bacteria in the CK, DPI, and Feces groups at the genus level. It can be seen that DPI can change the composition of gut microbiota, and the relative abundances of *Catenibacterium*, *Parabacteroides, Megamonas,* and *Clostridium* increased. The clustering tree showed the similarity and distribution characteristics of different gut microbiota in composition. Cluster analysis showed that there was a significant difference in gut microbiota between the CK group and the DPI group. In addition, Lefse analysis is one of the most commonly used methods for analyzing discrepancies in the abundance of gut microbiota. The branch diagram showed the difference in the intestinal microbial community structure and main bacterial groups between the two groups (Figure 8B). Gut microbiota with a higher relative abundance in different groups correspond to three different colored nodes in the diagram. Compared with the CK group, *Megamonas*, *Catenibacterium,* and *Parabacteroides* in the DPI group were the dominant bacteria. These results indicated that DPI could effectively change the gut microbial composition. Nevertheless, polysaccharides derived from different edible mushrooms exhibited different effects on the regulation of gut microbial composition. For example, *Flammulina velutipes* polysaccharides mainly increased the number of *Bifidobacterium* and *Bacteroidaceae* and reduced the numbers of *Lachnospiraceae* and *Enterococcaceae* [58]. *Pleurotus eryngii* polysaccharides mainly upregulated the abundance of *Firmicutes* [26]. *Tremella fuciformis* polysaccharides could promote the relative abundances of *Phascolarctobacterium*, *Bacteroides*, and *Lachnoclostridium* [10].

##### Functional Genes Prediction

**Figure 9:**
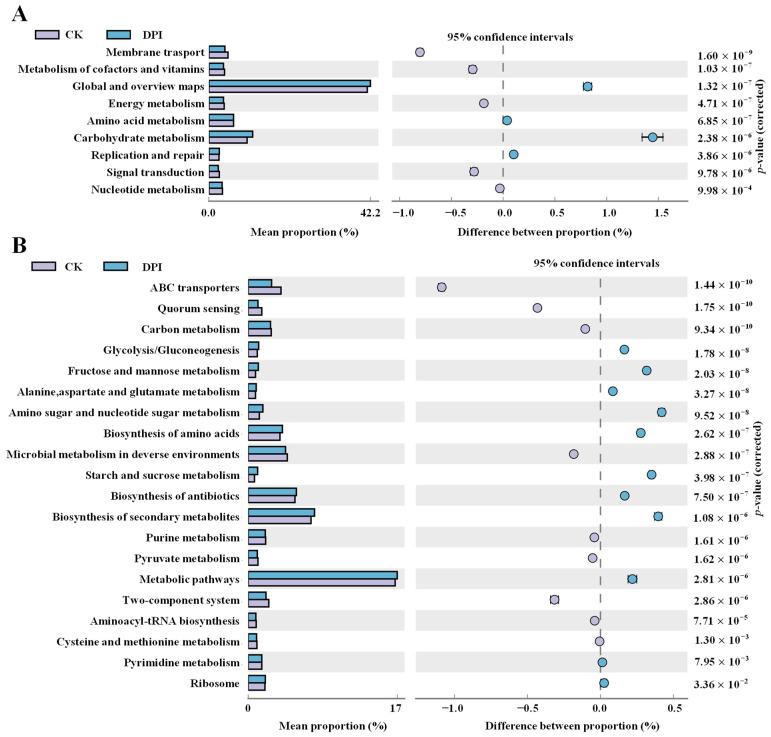
Differential analysis map of KEGG metabolic pathways at the second level (**A**) and third level (**B**) of classification.

The Kyoto encyclopedia of genes and genomes (KEGG) analysis was applied to predict the effect of DPI on functional metabolism following modulation of the gut microbiota. At level 2 (Figure 9A), the DPI could promote the “carbohydrate metabolism” and “global and overview maps” pathways, and more details presented in Level 3 (Figure 9B) indicated that “glycolysis/gluconeogenesis”, “fructose and mannose metabolism”, “alanine, aspartate and glucose metabolism”, “amino sugar and nucleoside sugar metabolism”, “biosynthesis of amino acids”, “Starch and sucrose metabolism”, “biosynthesis of antibiotics “, and “biosynthesis of secondary metabolites” were upregulated. It was predicted that DPI could promote the metabolic activity of gut microbiota and accelerate the metabolism of secondary products, amino acids, and antibiotics [59]. “Biosynthesis of secondary metabolites” is closely related to the “glycolysis/gluconeogenesis” pathway because the most common pathway to produce SCFAs in bacteria is through the glycolysis pathway [40]. The production of SCFAs is a highly complex and dynamic process. The researchers used radioisotope analysis to show that propionate was generated by a carbon dioxide fixation pathway while butyrate was most commonly formed by conventional acetyl-S coenzyme A condensation [60]. In addition, butyrate and propionate may be degraded into the smaller two carbon chain acetate by *Clostridium* species. Such interactions can involve the mutualistic production of SCFA [40]. Butyric acid is mostly absorbed by colon cells, while acetic acid and propionic acid can get into liver through the portal vein. Propionate is metabolized by hepatocytes, while acetic acid remains in the liver or is excreted into the peripheral venous system and then delivered to target organs by the circulatory system. They are not only energy sources but also key factors in gluconeogenesis and lipogenesis [20,40]. Therefore, it was speculated that DPI could regulate gut microbiota, affect energy homeostasis, and control energy storage and consumption [6].

## 4. Conclusions

This study found that DP was indigestible in the simulated saliva–gastrointestinal digestive system, indicating that DP was not destroyed by digestive juice and digestive enzymes and could arrive at the large colon smoothly. During in vitro microbial fermentation, the indigestible DP (DPI) was significantly decomposed and used by human gut microbiota, so as to regulate the composition of the microbial community. Specifically, DPI was able to promote the growth of *Bacteroides*, *Catenibacterium*, *Parabacteroides*, and *Megamonas* and inhibit the growth of detrimental bacteria. At the same time, DPI can induce the production of SCFAs during in vitro microbial fermentation, so as to change the acid–base environment of the large intestine. Collectively, the potential catabolic behavior of *D. indusiata* polysaccharide in the gastrointestinal tract was revealed in the present study. Nevertheless, studies that involve in vivo animal models should be carried out to further verify the catabolic behavior of *D. indusiata* polysaccharide.

## Figures and Tables

**Table 1:** Changes in reducing sugar content (C_R_) of *D. indusiata* polysaccharide during digestion and fermentation in vitro.

Processes	Time	C_R_ (mg/mL)
Salivary digestion	0 min	0.127 ± 0.011 ^a^
	2 min	0.130 ± 0.071 ^a^
	5 min	0.129 ± 0.002 ^a^
Gastric digestion	0 h	0.295 ± 0.011 ^a^
	0.5 h	0.284 ± 0.006 ^a^
	1 h	0.280 ± 0.022 ^a^
	2 h	0.252 ± 0.054 ^a^
Small intestinal digestion	0 h	0.789 ± 0.137 ^a^
	0.5 h	0.652 ± 0.153 ^a^
	1 h	0.619 ± 0.043 ^a^
	2 h	0.762 ± 0.127 ^a^
Microbial fermentation	0 h	0.194 ± 0.004 ^a^
	6 h	0.198 ± 0.015 ^a^
	12 h	0.179 ± 0.005 ^a^
	24 h	1.539 ± 0.002 ^b^
	48 h	2.585 ± 0.021 ^c^

Values represent mean ± standard deviation, and superscripts a–c differ significantly (*p* < 0.05) among different stages.

**Table 2:** Changes in fermentability (%), total polysaccharides content (%), and molecular weight (*M_w_*) of *D. indusiata* polysaccharide during digestion and fermentation in vitro.

	DP	DP-O	DP-G	DP-I	DPI-0 h	DPI-6 h	DPI-12 h	DPI-24 h	DPI-48 h
Fermentability	-	-	-	-	-	3.36 ± 0.91 ^d^	8.44 ± 0.48 ^c^	16.98 ± 0.29 ^b^	46.20 ± 1.05 ^a^
Total polysaccharides	96.53 ± 1.14 ^a^	95.57 ± 2.30 ^a^	95.49 ± 2.34 ^a^	95.33 ± 1.53 ^a^	95.01 ± 1.07 ^a^	91.82 ± 0.86 ^b^	88.99 ± 0.46 ^c^	80.88 ± 0.28 ^d^	51.12 ± 1.01 ^e^
*M_w_* (Da)									
Fraction 1 × 10^6^ Da	1.072 ± 0.037 ^a^	1.066 ± 0.041 ^a^	1.063 ± 0.036 ^a^	1.061 ± 0.036 ^a^	1.057 ± 0.029 ^a^	1.026 ± 0.032 ^ab^	0.982 ± 0.033 ^b^	0.851 ± 0.024 ^c^	0.595 ± 0.008 ^d^
Relative peak areas (%)		-	-	-	-	91.6	88.1	81.1	51.2
Fraction 2 × 10^5^ Da	-	-	-	-	-	1.014 ± 0.035 ^c^	1.062 ± 0.042 ^c^	1.519 ± 0.041 ^b^	2.028 ± 0.042 ^a^
Relative peak areas (%)	-	-	-	-	-	8.4	12.5	18.9	48.8

DP, *D. indusiata* polysaccharide; DP-O, DP-G, and DP-I indicate DP digested by oral digestion, oral –gastric digestion, and oral–gastrointestinal digestion, respectively; DPI-0 h, DPI-6 h, DPI-12 h, DPI-24 h, and DPI-48 h indicate indigestible *D. indusiata* polysaccharide (DPI) after microbial fermentation for 0, 6, 12, 24 and 48 h, respectively; values represent mean ± standard deviation, and superscripts a–e differ significantly (*p* < 0.05) among samples.

## Data Availability

Data are contained within the article.
